# Varieties of Ignorance: Mystery and the Unknown in Science and Religion

**DOI:** 10.1111/cogs.13129

**Published:** 2022-04-10

**Authors:** Telli Davoodi, Tania Lombrozo

**Affiliations:** ^1^ Wheelock College of Education and Human Development Boston University; ^2^ Department of Psychology Princeton University

**Keywords:** Ignorance, Mystery, Religion, Science, Unknown

## Abstract

How and why does the moon cause the tides? How and why does God answer prayers? For many, the answer to the former question is unknown; the answer to the latter question is a mystery. Across three studies testing a largely Christian sample within the United States (*N* = 2524), we investigate attitudes toward ignorance and inquiry as a window onto scientific versus religious belief. In Experiment 1, we find that science and religion are associated with different forms of ignorance: scientific ignorance is typically expressed as a personal unknown (“it's unknown to me”), whereas religious ignorance is expressed as a universal mystery (“it's a mystery”), with scientific unknowns additionally regarded as more viable and valuable targets for inquiry. In Experiment 2, we show that these forms of ignorance are differentially associated with epistemic goals and norms: expressing ignorance in the form of “unknown” (vs. “mystery”) more strongly signals epistemic values and achievements. Experiments 2 and 3 additionally show that ignorance is perceived to be a greater threat to science and scientific belief than to religion and religious belief. Together, these studies shed light on the psychological roles of scientific and religious belief in human cognition.

1

Even the most knowledgeable among us is surrounded by unknowns. A physicist is likely to believe that the universe was created by the big bang, without fully knowing how and why. A theist might believe that God answers prayers, without fully knowing how and why. And for those without scientific or religious expertise, ignorance is even more abundant: How and why does the moon affect the tides? How and why do bad things happen to good people? In the current paper, we investigate the role of ignorance across the domains of science and religion, focusing on ignorance regarding the answers to questions about *how* or *why* something is the case. When is this kind of ignorance taken as a basis to question belief (e.g., that the moon really does cause the tides, or that bad things really do happen to good people), and when is it a call to action––evidence that inquiry is worth pursuing? When is such ignorance considered a threat, and when is it better accepted, and even favored or revered?

Science and religion are sometimes characterized as different “ways of knowing” (e.g., Harris, 2007; see also Gould, 2002), suggesting that each domain involves a unique set of norms governing knowledge and justified belief. A complementary idea is that science and religion involve different forms of ignorance (or the *absence* of knowledge or belief), with unique sets of norms governing attitudes and practices concerning the unknown. This is the hypothesis we explore, with the expectation that attitudes toward ignorance have the potential to reveal epistemic commitments concerning the nature of inquiry and justified belief across domains, and also to shed light on the psychological roles of scientific and religious belief more broadly.

To test this hypothesis, we focus on ignorance concerning *how* and *why* something is the case––forms of knowledge often taken to be central to understanding (e.g., Lombrozo & Wilkenfeld, 2019). We refer to these manifestations of ignorance as “scientific unknowns” when they concern the natural world and canonically scientific content (e.g., how and why the big bang created the universe), and as “religious unknowns” when they incorporate the supernatural and concern canonically religious content (e.g., how and why God created the universe). We predict that scientific and religious unknowns differ in the following three respects: (1) scientific unknowns are regarded as more viable, appropriate, and valuable targets for inquiry than are religious unknowns; (2) scientific unknowns (by virtue of suggesting failures or limitations of inquiry) are perceived as more threatening to scientific belief and to the domain of science than religious unknowns are to religious belief and the domain of religion; and (3) these different attitudes toward ignorance are reflected in language, such that ignorance about scientific answers is more naturally expressed in terms of the unknown (e.g., “It's unknown how and why the big bang created the universe”), whereas ignorance about religious answers is more naturally expressed in terms of mystery (e.g., “It's a mystery how and why God created the universe”).

Prior work suggests that scientific and religious beliefs may indeed differ with respect to the epistemic practices and norms that govern them. For instance, there is evidence that scientific (vs. religious) beliefs are more likely to be justified by appeal to evidence (Metz, Weisberg, & Weisberg, 2018; Shtulman, 2013), more likely to be held with high confidence (Cui et al., 2020; Davoodi et al., 2019; Harris, Pasquini, Duke, Asscher, & Pons, 2006), and more likely to be perceived as objectively true (Heiphetz, Spelke, Harris, & Banaji, 2013; see also Friesen, Campbell, & Kay, 2015; Gottlieb, 2007; Heiphetz, Spelke, Harris, & Banaji, 2014). Consistent with the idea that people intuitively differentiate scientific and religious belief, Heiphetz, Landers, and Van Leeuwen (2018) found that English‐speaking adults in the United States tend to use the word “think” in discussing scientific claims (e.g., “I *think* the universe started with the Big Bang”) and the word “believe” in discussing religious claims (e.g., “I *believe* God created the world in seven days”). Evidence for a similar distinction has been documented across several other languages and cultures (Van Leeuwen, Weisman, & Luhrmann, 2021).

These findings fit within a developing framework according to which scientific and religious beliefs are tuned to different psychological functions (e.g., Davoodi & Lombrozo, 2021; Davoodi & Lombrozo, 2020; Tetlock, 2002; Van Leeuwen, 2014). For instance, Davoodi and Lombrozo (2021) find that scientific explanations are more strongly associated with epistemic merits (e.g., being logical and based on evidence), whereas religious explanations are more strongly associated with nonepistemic merits (e.g., social, emotional, and moral benefits). One possibility, then, is that scientific beliefs typically serve more epistemic roles––such as offering veridical representations of the world that support accurate predictions and effective interventions––whereas religious beliefs typically serve other roles, such as buffering existential anxiety, signaling group membership, or promoting prosociality within groups (e.g., Norenzayan, 2013; Norenzayan & Hansen, 2006; Pichon, Boccato, & Saroglou, 2007). If these functional profiles predict different attitudes toward scientific versus religious belief, we should expect corresponding differences in attitudes toward scientific versus religious ignorance.

What might this functional approach predict about attitudes toward scientific versus religious ignorance? First, consider the case of science. If science (and scientific belief) is aligned with epistemic goals, then ignorance should be acknowledged because it defines the contours of prior success and points the way toward future progress. It might be aversive (insofar as it indicates current limitations), but it should also be motivating––a sign that there is further inquiry to pursue. This is consistent with the observation that recognizing and reporting ignorance and uncertainty is central to scientific practice (see Smithson, 1993), but empirical evidence on the corresponding psychology has been relatively sparse.

Prior work has documented variability in public attitudes toward scientific uncertainty (Gustafson & Rice, 2020), as well as preferences for calibrated expressions of uncertainty concerning factual claims (e.g., Sah, Moore, & MacCoun, 2013; Tenney, MacCoun, Spellman, & Hastie, 2007). However, little is known about scientific ignorance as such. Kominsky, Langthorne, and Keil (2016) found that adults (and even children as young as 9‐years‐old) appreciate “virtuous ignorance” when it comes to factual matters that are actually or practically unknowable, such as the number of leaves on all the trees in the world. In these studies, participants favored an informant who acknowledged ignorance about “unknowable” facts over an informant who claimed to have knowledge. Similarly, preschoolers have been shown to distinguish between an informant who professes ignorance and an informant who provides inaccurate information, attributing and endorsing future knowledge more often to the informant in the former instance (Kushnir & Koenig, 2017). This research suggests that recognizing (scientific) ignorance is likely to be valued in certain contexts. However, it remains unclear whether such ignorance has implications for inquiry. Inquiry concerning something “knowable” may be fruitful, but inquiry into the unknowable may be a waste of time.

What about ignorance in the case of religion? At least some religious traditions, including several Christian traditions, seem to embrace a notion of *mystery*, perhaps signaling that something is not only unknown, but also unknowable, or otherwise inappropriate as a target of inquiry. Monsignor Charles Pope (2013), for example, states that “in the ancient Christian tradition, mystery is something to be accepted and even appreciated…the attempt to solve many of the mysteries in the Christian tradition would be disrespectful, and prideful too.” This attitude toward the unknown is potentially puzzling if religion (and religious belief) is regarded as a predominantly epistemic exercise. However, it makes more sense if religion (and religious belief) is instead aligned with other goals––such as signaling commitment or structuring community, and to that end nurturing faith and humility.

Consistent with these suggestions, there is evidence that at least within some populations, religion (vs. science) is in fact judged to be less oriented toward inquiry and more oriented toward mystery. In one set of studies, predominantly Christian participants within the United States judged that questions about science demanded an explanation more strongly than questions about religion, and correspondingly, that it was more appropriate to answer questions about religion with “It's a mystery” than it was to answer questions about science in the same way (Liquin, Metz, & Lombrozo, 2020). These results were driven in part by participants’ stronger beliefs that questions about religion (vs. science) *could not* be answered (because the answer is beyond human comprehension), but also that they *should not* be answered (perhaps because doing so would be seen as disrespectful or prideful, as Monsignor Charles Pope suggests). In other studies (Gill & Lombrozo, 2019), a similar population was presented with vignettes about a character who encounters a scientific or religious claim for the first time, and decides to either pursue further inquiry (i.e., seeking evidence or explanation), or abdicates from further inquiry (i.e., does not seek further evidence or explanation). Participants were asked to judge how committed that character is to both science and religion. Characters who pursued inquiry (on any topic) were judged more committed to science than characters who did not. By contrast, for religious claims in particular, characters who pursued further inquiry were regarded as *less* committed to religion than were characters who chose not to pursue further evidence or explanation.

The theoretical considerations and evidence just reviewed motivate our predictions that scientific unknowns will be regarded as more viable, appropriate, and valuable targets for inquiry than religious unknowns, and that the latter will be more strongly associated with “mystery” versus a more generic expression of ignorance, such as “unknown.” These considerations also suggest that whereas scientific ignorance may be regarded as more circumscribed in scope (limited to particular people or points in time), religious ignorance might be seen as insurmountable: not merely a current unknown, but something that is in principle unknowable, and appropriately so.

We test this suite of predictions in Experiments 1 and 2. In Experiment 1, we investigate the expressions of ignorance that participants judge most appropriate in response to questions about science and religion (e.g., how and why the big bang occurred vs. how and why God answers prayers). We predict that expressions of ignorance in science will tend to focus on the unknown (vs. mystery) and to involve a personal scope (“it's unknown to me”), whereas expressions of ignorance within religion will tend to focus on mystery (vs. the unknown) and a universal scope (“it's a mystery [to everyone]”). In Experiments 1 and 2, we additionally investigate whether scientific unknowns, as compared to religious mysteries, are seen as more consistent with epistemic goals, such that they are more viable, appropriate, and valuable targets for inquiry (Experiment 1), and more indicative of epistemic values and norms (Experiment 2).

In Experiments 2 and 3, we additionally consider the implications of ignorance for belief. If someone learns that it is unknown how the first living organisms emerged from natural processes, or that it is a mystery how God parted the Red Sea, is this likely to threaten the corresponding beliefs––that the first living organisms emerged from natural processes, or that God parted the Red Sea? Do scientific unknowns pose a threat to science, or religious mysteries to religion? Does the level of threat to a belief or domain depend on the form that ignorance assumes: a mere *unknown* versus a *mystery*? If science is evaluated in terms of its epistemic success, then ignorance might be threatening insofar as it suggests the domain of science is circumscribed (because something is unknowable) or that a belief system is incomplete (because something is currently unknown). On the other hand, if religious ignorance is assumed to be inevitable or even desirable, religious ignorance is unlikely to threaten the corresponding beliefs or the domain as a whole.

Prior work offers conflicting predictions. Klein and Colombo (2018) develop a theoretical account of mysteries, from which they argue that learning that something is a mystery can sometimes offer evidence against the corresponding belief. Specifically, they suggest that some mysteries pose a conflict with our pre‐existing beliefs (e.g., Jesus turning water into wine conflicts with our intuitive theories of matter and material change), while the negation of the mystery does not (that Jesus did not turn water into wine). They argue that in such cases, learning that something is a mystery offers some evidence against the claim itself (that Jesus in fact turned water into wine). Because both religious and scientific claims are often counterintuitive (Boyer, 1994; 2001; Boyer & Ramble, 2001; Lane & Harris, 2014), Klein and Colombo's analysis suggests that learning that something is a mystery is potentially threatening to beliefs from both domains (but see Bussey (2011) for an argument about mystery as an appropriate form of not knowing in both science and religion, which suggests that mysteries may not be threatening to either domain).

On the other hand, there is evidence that religious beliefs may derive psychological value from their status as unverifiable. In particular, Friesen et al. (2015) found that religious believers reported greater religious conviction after reading a passage that claimed the existence of God could never be proven or disproven, versus one that claimed the existence of God would eventually be proven or disproven. Moreover, when their religious beliefs were threatened, participants were more likely to endorse unfalsifiable (vs. falsifiable) reasons for religious beliefs. If “mystery” signals *unverifiability*, a declaration of mystery could actually bolster, rather than challenge, the corresponding religious belief.

In Experiment 2, we test the hypothesis that ignorance is more threatening to scientific belief and to science than to religious belief and religion, with the largest threat coming from ignorance in the form of mystery (because it is less consistent with epistemic norms). In Experiment 3, we look at personal belief, asking whether people's confidence in their own beliefs decreases in the face of stated ignorance, with differential effects across domains (science vs. religion) and forms of ignorance (unknown vs. mystery). Across Experiments 1–3, we thus offer the first systematic investigation of the psychology of scientific and religious ignorance, including implications for inquiry and belief.

## 2 Experiment 1

In Experiment 1, we investigated the forms of ignorance associated with scientific versus religious explanation‐seeking questions (e.g., how and why humans evolved from earlier primates vs. how and why God answers prayers). We varied two dimensions of ignorance: *unknown* (“it's unknown”) versus *mystery* (“it's a mystery”), and *universal* (e.g., “it's unknown”) versus *personal* (e.g., “it's unknown to me”). We predicted that compared to science, religion would be more strongly associated with *mystery* versus *unknown*, and with *personal* versus *universal*.

To create conditions in which participants would naturally respond to a “how and why” question about science or religion with some form of ignorance, participants were first asked to report religious or scientific beliefs that they hold (e.g., that humans evolved from earlier primates, or that God answers prayers), but for which they do not know the “how and why.” They were then asked to select the most appropriate response from the four options generated by crossing *unknown* versus *mystery* with *personal* versus *universal*.

In addition, participants were asked about the possibility and value of inquiry into the “how and why” concerning their belief (e.g., “It would be fruitful to investigate how this happens”), the norms governing inquiry (“People shouldn't try to answer how and why this happens”), the verifiability of their belief (e.g., “This belief can be tested”), and whether it was held on faith (“I hold this belief on faith”). Most of these items were included to offer a conceptual replication of prior work documenting perceived differences between science and religion, with science more strongly associated with inquiry (e.g., Gill & Lombrozo, 2019; Liquin et al., 2020) and with epistemic dimensions of belief (e.g., Davoodi & Lombrozo, 2021). Following up on this domain distinction, the current study allowed us to ask whether *unknown* reflects a more “scientific” profile than *mystery* with respect to these aspects of inquiry and belief.

The procedures, predictions, and analyses for Experiment 1 were preregistered and are available on OSF at (https://osf.io/43yte/). A copy of the survey and data are available at (https://osf.io/8x6vq/).

## 3 Methods

### 3.1 Participants

Participants were 506 adults recruited on Prolific (257 self‐identified as a woman, 241 as a man, and 8 as nonbinary, *M*_Age_ *=* 34 years, *SD*_Age_ = 12 years). Of these, 37% identified as Christian and 29% as Atheist, with the remaining 34% including “other” (14%), “Spiritual” (11%), and other religious affiliations (9%)––Buddhist, Jewish, Hindu, Muslim, and combinations of two or more affiliations. Participation in all studies was restricted to Prolific workers in the United States who had not participated in any related pilot studies, and who had an approval rating of at least 95% based on at least 100 prior tasks. An additional 103 participants were excluded from analyses because they did not meet criteria for belief generation in either domain (i.e., religious or scientific beliefs; *N* = 99), as detailed below, and/or because they did not pass attention checks in blocks where they did meet criteria for belief generation.

### 3.2 Procedure

Participants completed all procedures online using Qualtrics Survey Software (see OSF page for the full survey). Each participant first consented to participate and pledged to pay attention and respond carefully. Participants were then told: “In this survey, we will ask you about some of your beliefs. Sometimes, we may hold a belief (for example, that humans evolved from earlier primates), but when asked about the details of this belief (for example, how and why humans evolved from earlier primates), we may not have all the information. These are the kinds of beliefs we would like to ask you about.” They then completed a brief training on the “content” of beliefs. This was to ensure that when asked to describe their belief, participants only produced the propositional content of their belief (e.g., “humans evolved from earlier primates” vs. “I believe that humans evolved from earlier primates”), as this content was used for later questions. In the training phase, they were also familiarized with the scale used for subsequent questions.

On the next page, participants were asked to indicate whether they could think of a scientific belief or a religious/spiritual belief (order counterbalanced) that they hold, but for which they do not know much about the how and why. If they indicated that they could not do so, they were then asked the same question about a belief from the other domain. If they indicated that they could not do so in the second domain either, they were taken to the end of the survey and corresponding data were excluded from analysis.

If participants indicated that they *could* think of a belief that met our requirements in the given domain, they were invited to type the content of the belief into a text box. The text entered into this box was then used on the next page, where participants were asked: “Which of the following answers do you think is most appropriate in response to the question of how and why [generated belief].” Using a drop‐down menu, participants selected one of: “It's unknown,” “It's unknown to me,” “It's a mystery,” “It's a mystery to me” (order randomized).

On the next page, participants rated their agreement with five statements about the value and possibility of inquiry regarding their belief (order randomized), on a scale from –3 (strongly disagree) to 3 (strongly agree), with 0 representing “neither agree nor disagree” (see Table 1, items 1–5). On the next page, participants rated their agreement with four statements about the evidential status of the belief itself (see Table 1, items 6–9). If participants reported holding a belief in the second domain that met our requirements, these steps were repeated for the second belief. There was one attention check item intermingled among the statements about belief for each domain (e.g., “for this item, please select the midpoint of the scale.”). If participants failed the attention check for a given domain, data from that domain were excluded from analysis.

**Table 1:** Experiment 1––Measures used to assess judgments about mechanism and belief

Questions about inquiry	
1. *Inquiry‐how*	“It would be fruitful to investigate how this happens.”
2. *Inquiry‐why*	“It would be fruitful to investigate why this happens.”
3. *Inquiry‐investigation*	“Investigating this phenomenon should be a priority for future research.”
4. *Epistemic limit*	“How and why this happens is beyond human comprehension.”
5. *Epistemic regulation*	“People shouldn't try to answer how and why this happens.”
Questions about belief	
6. *Verifiability‐verified*	“This belief can be verified as true or false.”
7. *Verifiability‐tested*	“This belief can be tested.”
8. *Verifiability‐evidence*	“In principle, one could find evidence relevant to whether this belief is true or false.”
9. *Faith*	“I hold this belief on faith.”

At the end of the survey, participants completed a brief demographics survey where they were asked about gender, age, income, education, religiosity, and religious affiliation.

## 4 Results

We first verified that participants who reported scientific or religious beliefs that met our requirements in fact generated propositions from the corresponding domains. In particular, we did not want to code beliefs that rejected religion as religious (e.g., “heaven or hell does *not* exist”), or pseudoscientific beliefs as scientific (e.g., “I believe there really is a big‐foot”). Two independent coders coded all beliefs as “domain appropriate” or not, with very high levels of agreement (religious beliefs: 98% agreement; *kappa* = 0.84, *SE* = 0.06, *p* < .001, *CI* [0.71, 0.95]; scientific beliefs: 99% agreement; *kappa =* 0.84, *SE* = 0.08, *p* < .001, *CI* [0.67, 0.99]). Disagreements were resolved by a third coder. This resulted in excluding 22 beliefs as domain‐inappropriate for religion (vs. 274 as domain‐appropriate and included in analyses of all qualifying beliefs), and 11 as domain‐inappropriate for science (vs. 431 as domain‐appropriate and included in analyses of all qualifying beliefs). However, including these beliefs in the analyses that follow does not change the patterns reported in the results.

Domain‐appropriate religious beliefs mostly reflected canonical beliefs from Abrahamic traditions (e.g., God's creation of the universe/humans; God's existence; the existence of an afterlife in some form) as well as Christian beliefs about Jesus’ life and crucifixion. Spiritual beliefs included beliefs in karma and reincarnation, and were coded as domain‐appropriate together with religious beliefs. Domain‐appropriate scientific beliefs were more diverse compared to religious beliefs and included a range of scientific topics, including climate change, physical health, mental health, genetics, evolution, and the big bang. A complete list of all domain‐appropriate and domain‐inappropriate beliefs generated by participants is included on OSF (https://osf.io/h9zk3/).

### 4.1 Analytic approach

We addressed our three research questions with analyses (detailed below) conducted in two ways. We first analyzed all qualifying beliefs, whether or not the participants generated a belief from a single domain or from both domains. We then repeated each analysis including only data from participants who generated a qualifying belief in *both* domains (*N* = 205). The advantage of the former approach is that it excludes fewer participants; the advantage of the latter is that it ensures that any effects, if found, reflect genuine differences across domains, and not selection effects leading some kinds of participants to be systematically excluded from a single domain. All reported confidence intervals refer to the 95% level.

### 4.2 Forms of ignorance in science and religion

Our first research question concerned the relationship between domain (religion vs. science) and forms of ignorance, which varied along two dimensions (unknown = 1 vs. mystery = 0, and individual scope = 1 vs. universal scope = 0). For each dimension, we regressed the response code (0 vs. 1) on domain using a mixed‐effects binomial regression using the *glmer* function from the *lme4* package in *R*. We also included by‐participant intercepts to account for the within‐subjects structure of domain.

**Fig. 1:**
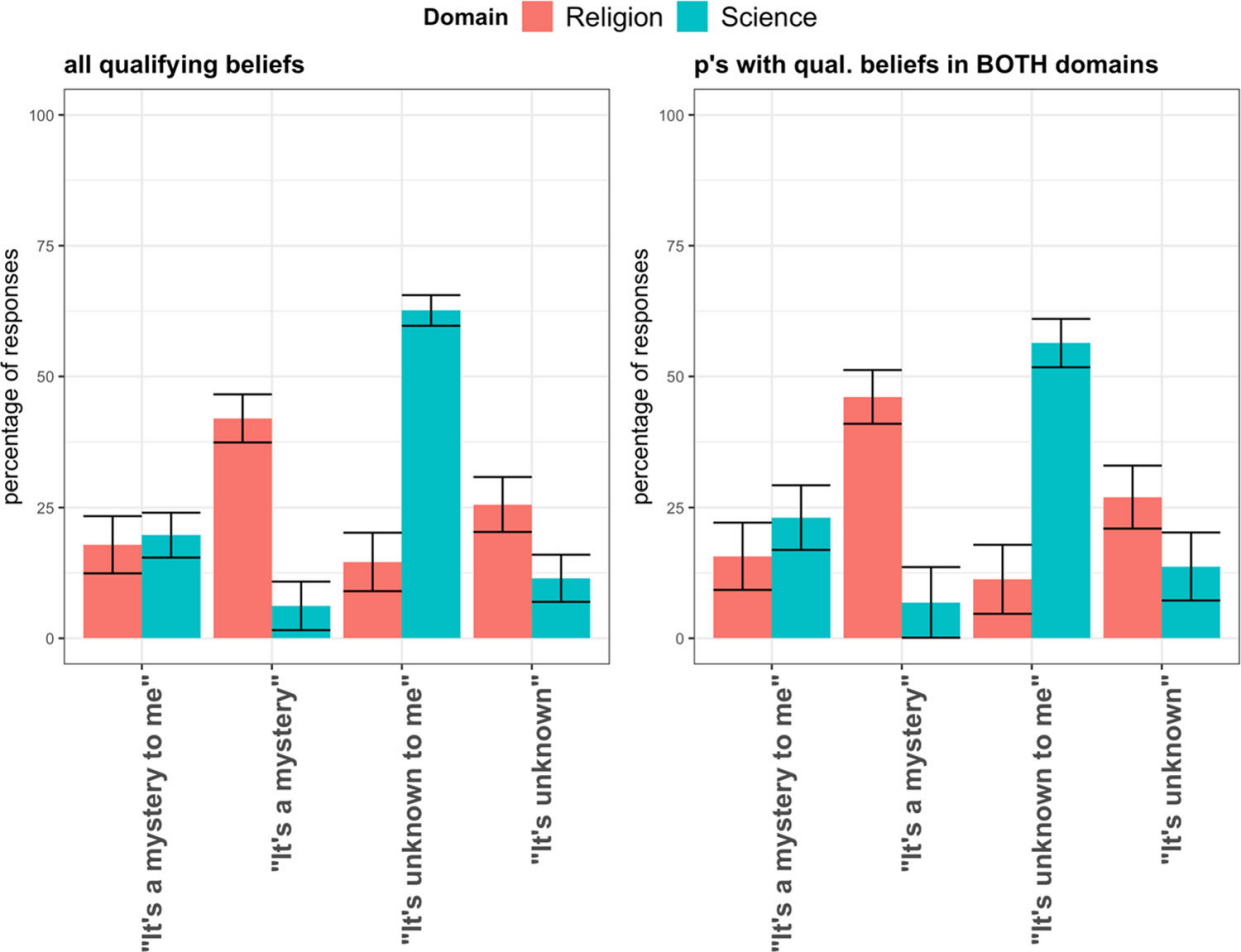
Experiment 1––Percentage of responses corresponding to each Ignorance Type and Ignorance Scope as a function of Domain for all qualifying beliefs (left) and for participants with qualifying beliefs in both domains (right). Note: Error bars represent +/–1 standard error of the sample proportions.

For ignorance type, we found that questions about scientific beliefs were more often judged to be “unknown” and less often judged to be “a mystery” than were questions about religious beliefs (*B* = 1.49, *SE* = 0.18, *p* < .001, *OR* = 4.45, *CI* [3.08, 6.42]). When considering only participants with qualifying beliefs in both domains, we replicated these results (*B* = 1.38, *SE* = 0.23, *p* < .001, *OR* = 3.97 *CI* [2.82, 6.26] (Fig. 1).

For the scope of ignorance, we found that questions about scientific beliefs were more often judged to involve personal ignorance and less often judged to involve universal ignorance than were questions about religious beliefs (*B* = 2.45, *SE* = 0.30, *p* < .001, *OR* = 11.57, *CI* [6.48, 20.67]). When considering only participants with qualifying beliefs in both domains, we replicated these results (*B* = 2.53, *SE* = 0.35, *p* < .001, *OR* = 12.61, *CI* [6.32, 25.14] (Fig. 1).

### 4.3 Attitudes toward inquiry and evidence in science and religion

**Fig. 2:**
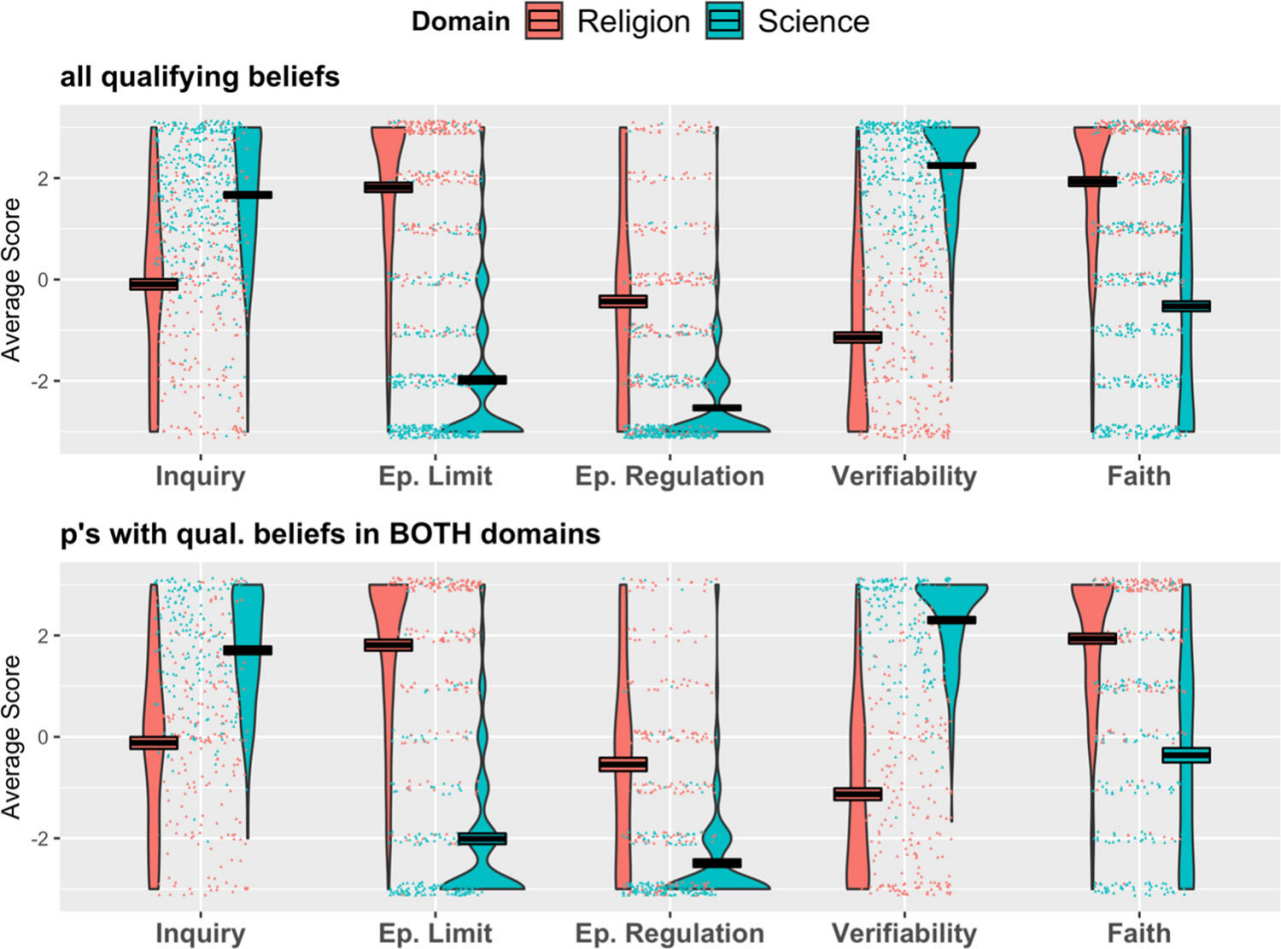
Experiment 1––Average ratings of epistemic values and faith as a function of Domain for all qualifying beliefs (upper panel) and for participants with qualifying beliefs in both domains (lower panel). Note: “0” represents “neither agree nor disagree.” Points represent jittered data points. Boxes represent average scores––black line in the middle––and +/– 1 SEM.

Our second research question concerned the perceived roles of inquiry, verifiability, epistemic regulation, epistemic limits, and faith across domains. We first created composite scores for inquiry and verifiability. Based on pilot testing, we expected the three inquiry items to form a reliable construct, which warranted combining them into a single score (*α* = 0.87). Likewise, the three items measuring verifiability formed a reliable construct and were combined into a single score (*α* = 0.94). For each dependent variable, we conducted mixed‐effects linear regression analyses with Domain as a predictor, using the *lme* function from the *nlmer* package in *R*. Again, we included by‐participant random intercepts to account for variability within participants across Domain. Fig. 2 shows the effects of Domain for each measure.

For inquiry, we found that questions about science were judged more valuable targets of inquiry than questions about religion (*B* = 1.76, *SE* = 0.11, *t* = 16.50, *p* < .001, *CI* [1.55, 1.97]). We replicated this result when only participants with qualifying beliefs in both domains were included (*B* = 1.82, *SE* = 0.14, *t* = 13.24, *p* < .001, *CI* [1.82, 2.10]).

For epistemic limits and regulation, we found that questions about science were less likely to be judged beyond human comprehension than were questions about religion (*B* = –3.80, *SE* = 0.12, *t* = –32.52, *p* < .001, *CI* [–4.03, –3.57]), and that participants were more likely to disagree that scientific questions should not be pursued than that religious questions should not be pursued (*B* = –2.10, *SE* = 0.11, *t* = –19.41, *p* < .001, *CI* [–2.31, –1.88], respectively). Both of these results replicated when only participants with qualifying beliefs in both domains were included (*B* = –3.82, *SE* = 0.15, *t* = –25.18, *p* < .001, *CI* [–4.12, –3.52]; *B* = –1.95, *SE* = 0.15, *t* = –12.81, *p* < .001, *CI* [–2.25, –1.67], respectively).

For verifiability, we found that scientific beliefs were rated as more verifiable than religious beliefs, both when all qualifying beliefs were included (*B* = 3.39, *SE* = 0.10, *t* = 34.80, *p* < .001, *CI* [3.20, 3.58]) and when only participants with qualifying beliefs in both domains were included (*B* = 3.43, *SE* = 0.13, *t* = 26.01, *p* < .001, *CI* [3.17, 3.69]).

Finally, for faith, we found that scientific beliefs were less faith‐based compared to religious beliefs, both when all qualifying beliefs were included (*B* = –2.44, *SE* = 0.13, *t* = –18.03, *p* < .001, *CI* [–2.71, –2.17]) and when participants with qualifying beliefs in both domains were included (*B* = –2.30, *SE* = 0.16, *t* = –14.14, *p* < .001, *CI* [–2.62, –1.98]).

### 4.4 Associations between ignorance and attitudes toward inquiry and evidence within domains

The analyses above reveal that on average, questions about science versus religion are associated with different forms of ignorance, and also with different attitudes toward inquiry and evidence. Our third research question concerned whether variation in forms of ignorance was associated with these attitudes *within* each domain, with *unknown* showing a more scientific profile than *mystery*. Correspondingly, we conducted linear regression models within each domain on each of the four epistemic measures and on “faith,” with either Ignorance Type (unknown vs. mystery) or Ignorance Scope (personal vs. universal) as a predictor.

Within the domain of religion, we did not find significant effects of ignorance type nor of ignorance scope on inquiry (Ignorance Type: *B* = –0.13, *SE* = 0.21, *t* = –0.63, *p* = .53; Ignorance Scope: *B* = 0.30, *SE* = 0.22, *t* = 1.40, *p* = .16), epistemic limits (Ignorance Type: *B* = –0.09, *SE* = 0.19, *t* = –0.48, *p* = .63; Ignorance Scope: *B* = –0.18, *SE* = 0.20, *t* = –0.91, *p* = .36), epistemic regulation (Ignorance Type: *B* = –0.08, *SE* = 0.23, *t* = –0.34, *p* = .73; Ignorance Scope: *B* = –0.20, *SE* = 0.24, *t* = –0.84, *p* = .40), or faith‐based belief (Ignorance Type: *B* = –0.26, *SE* = 0.18, *t* = –1.50, *p* = .13; Ignorance Scope: *B* = –0.28, *SE* = 0.18, *t* = –1.52, *p* = .13). For verifiability, the effect of Ignorance Type also failed to reach significance (*B* = –0.14, *SE* = 0.20, *t* = –0.70, *p* = .48), but there was a significant effect of Ignorance Scope (*B* = 0.48, *SE* = 0.21, *t* = 2.27, *p* = .02, *CI* [0.06, 0.90]): personal (vs. universal) ignorance was associated with higher attributions of verifiability to religious beliefs. All of these patterns remained consistent when we restricted our sample to only those with qualifying beliefs in both domains (note that in this second set of models, we analyzed participants' *religious* beliefs, but only for those whose generated qualifying beliefs in both domains).

Within the domain of science, Ignorance Type was not significantly associated with inquiry (*B* = –0.04, *SE* = 0.13, *t* = –0.29, *p* = .77), epistemic limits (*B* = –0.21, *SE* = 0.16, *t* = –1.30, *p* = .20), epistemic regulation (*B* = –0.06, *SE* = 0.11, *t* = –0.57, *p* = .57), or verifiability (*B* = 0.04, *SE* = 0.10, *t* = 0.44, *p* = .70). However, Ignorance Scope was significantly associated with these measures, such that universal (vs. personal) ignorance was more strongly associated with higher ratings for inquiry (*B* = –0.37, *SE* = 0.15, *t* = –2.48, *p* = .01, *CI* [–0.65, –0.07]) and epistemic limits (*B* = –1.16, *SE* = 0.18, *t* = –6.37, *p* < .001, *CI* [–1.52, –0.80]), and lower ratings for verifiability (*B* = 0.54, *SE* = 0.11, *t* = 4.74, *p* < .001, *CI* [0.31, 0.76,]). Ignorance Scope did not predict ratings for epistemic regulation (*B* = –0.19, *SE* = 0.12, *t* = –1.53, *p* = .12). All of these patterns remained the same when restricting the sample only to participants with qualifying beliefs in both domains (note that in this second set of models, we analyzed participants' scientific beliefs, but only for those whose generated qualifying beliefs in both domains).

Finally, ratings for the faith‐based item in the domain of science were the only dimension to show significant effects of Ignorance Type: mystery (vs. unknown) was associated with stronger agreement that a belief was held on faith (*B* = –0.57, *SE* = 0.22, *t* = –2.57, *p* = .01, *CI* [–1.00, –0.13]). Ignorance Scope also significantly predicted “faith‐based” ratings, such that universal (vs. personal) ignorance was associated with stronger agreement that a belief was held on faith (*B* = –0.59, *SE* = 0.25, *t* = –2.33, *p* = .02, *CI* [–1.09, 0.09]). While the effect of Ignorance Scope remained significant for participants with qualifying beliefs in both domains, the effect of Ignorance Type was not significant within this sample (*B* = –0.28, *SE* = 0.31, *t* = –0.92).

## 5 Discussion

The findings from Experiment 1 support the prediction that unknown answers to scientific and religious questions are associated with different forms of ignorance. Scientific questions about how and why something is the case were most often answered with “it's unknown to me,” while religious questions about how and why something is the case were most often answered with “it's a mystery.” These responses reveal variation along two dimensions of ignorance: *unknown* versus *mystery*, and *personal* versus *universal* scope.

Experiment 1 also corroborated prior research (Davoodi & Lombrozo, 2020; Gill & Lombrozo, 2019; Liquin et al., 2020) in finding reliable differences across domains in attitudes toward inquiry and evidence. Specifically, we found that compared to scientific beliefs, religious beliefs were judged less appropriate targets for inquiry, were deemed less verifiable, and were more likely to be held on faith. Interestingly, this held even though participants were ignorant of the “how and why” regarding their beliefs in *both* domains.

We also predicted that even within a domain, selecting *unknown* versus *mystery* as the appropriate form of ignorance would be associated with a more “scientific” profile. This prediction was only borne out when it came to holding a belief on faith within the domain of science: participants who selected “it's a mystery [to me]” (vs. “it's unknown [to me]”) in response to a scientific question were more likely to indicate that their belief was held on faith, although this difference did not persist among participants with qualifying scientific *and* religious beliefs. We did find more reliable differences between *personal* versus *universal* ignorance: within both domains, universal ignorance was associated with lower ratings for verifiability, and within science, universal ignorance was also associated with higher ratings for inquiry, epistemic limits, and being held on faith.

## 6 Experiment 2

Experiment 1 successfully demonstrated that science and religion are associated with different forms of ignorance: *unknown* in science, and *mystery* in religion. Experiment 1 was also successful in corroborating prior work on differences across domains, with scientific claims more strongly associated with evidence and inquiry than religious claims. Experiment 2 went beyond these domain‐based associations by introducing an experimental manipulation of ignorance: participants were presented with a hypothetical expert who was posed a question about science or religion for which the participant did not know the “how and why,” and to which the expert responded with either “It's unknown” or “It's a mystery.” Although this is a very subtle manipulation, we expected participants to draw different inferences about the expert as a function of their ignorance type, and for ignorance to be treated differently across domains. We elaborate on both predictions below.

Our first hypothesis concerned the epistemic commitments reflected by *unknown* versus *mystery*. Based on the material reviewed in the introduction and the findings from Experiment 1, we predicted that expressing ignorance in the form of *unknown* (vs. *mystery*) would be more consistent with a scientific orientation, and thus with epistemic goals (e.g., wanting to know more), epistemic achievements (e.g., being knowledgeable), and epistemic norms (e.g., valuing truth). To test this, participants were asked to indicate the extent to which the expert who reported “It's unknown” or “It's a mystery” is curious, knowledgeable, and values truth.

Our second hypothesis concerned the implications of ignorance in each domain. Does ignorance in response to a scientific question (e.g., stating that it is unknown or a mystery how and why the universe was caused by the big bang) threaten the truth of that belief (e.g., that the universe was so created) or science as a whole? Does ignorance in response to a religious question (e.g., stating that it is unknown or a mystery how and why God created the universe) threaten the truth of that belief or religion as a whole? We expected that in the domain of science, *mystery* would be more threatening than *unknown*, because it is less consistent with the epistemic norms that govern science. For the domain of religion, by contrast, we expected this effect to be attenuated or reversed.

The procedures, predictions, and analyses for Experiment 2 were preregistered and are available in OSF at (https://osf.io/6daec/). A copy of the survey and data are available at (https://osf.io/pb3xk/).

## 7 Methods

### 7.1 Participants

Participants were 1014 adults recruited on Prolific (559 self‐identified as a woman, 443 as a man, and 12 as nonbinary, *M*_Age_ *=* 33 years, *SD*_Age_ = 12 years). Of these, 39% identified as Christian and 24% as Atheist, with the remaining 37% including “other” (14%), Spiritual (11%), and other religious affiliations––Buddhist, Jewish, Hindu, Muslim––as well as combinations of two or more affiliations (12%). Participation in all studies was restricted to Prolific workers in the United States who had not participated in any related pilot studies, and who had an approval rating of at least 95% based on at least 100 prior tasks. An additional 110 participants were excluded from analyses because they did not meet criteria for belief generation in either domain (i.e., religious or scientific beliefs; *N* = 92), as detailed below, and/or because they did not pass attention checks in blocks where they did meet criteria for belief generation.

### 7.2 Procedure

Participants completed all procedures online using Qualtrics Survey Software (see OSF page for the survey). Each participant first consented to participate and pledged to pay attention and answer questions carefully. The task was introduced as follows: “In this survey, we will ask you several questions. For most of them, there are no right or wrong answers. We ask that you try your best to answer the questions based on your beliefs and, where relevant, the information provided.”

#### 7.2.1 Phase 1––Belief identification

For each participant, we first identified a proposition that met the requirements outlined in Experiment 1 (namely, one that the participant endorses, but for which they do not know much about the how and why). To do so, participants were presented with claims from the domain of religion or science, with each domain presented in a separate block in counterbalanced order. For each domain, participants worked through a minimum of 1 and a maximum of 5 claims in a fixed order until we identified one for which (1) the participant indicated that they believed that claim, and (2) when asked whether they “know how and why this happens/d?” they indicated “no” (see Table 2 for a complete list of religious and scientific claims). As soon as a belief that met the relevant requirements was identified, participants moved on to the next phase. If a belief that met both requirements was not identified after the fifth claim, we did not analyze data from that participant for that domain.

**Table 2:** Experiment 2––Questions presented to participants from each domain in the belief identification phase

	Religion	Science
1	Do you believe that God answers prayer?	Do you believe that CO2 emissions are causing climate change?
2	Do you believe that the soul leaves the body after death?	Do you believe that the moon causes tides?
3	Do you believe that events in this world are sometimes caused by divine intervention?	Do you believe that the universe was caused by the big bang?
4	Do you believe that some form of life continues after death?	Do you believe that physical exercise rejuvenates cells in the brain?
5	Do you believe that God created the universe?	Do you believe that humans evolved from earlier forms of primates?

Note that each participant could see 1–5 of these questions, depending on which met the requirements of belief without knowledge of “how and why.”

#### 7.2.2 Phase 2––Expert introduction and ignorance affirmation

After identifying a belief that met our requirements, we introduced participants to an expert who was said to hold the same belief that met our requirements for the participant. For example, if a participant indicated that they believed God answers prayers but did not know much about the “how and why,” they were told: “‘A’ is an expert when it comes to religious questions like those you were asked about. ‘A’ also believes that God answers prayer.” Participants were then told “When asked how and why this happens, here is what ‘A’ said,” with the response being either “It's a mystery” or “It's unknown.” This manipulation was between‐participants, with random assignment.

#### 7.2.3 Phase 3––Ratings

Next, participants indicated their agreement with three sets of four statements: belief‐threat items, domain‐threat items, and expert‐epistemic items (Table 3). The set of expert‐epistemic items included one attention check item intermingled among the other three questions. Each set of items was presented in a single block, with both block order and the order of items within each block randomized. Items were rated on a scale from –3 (strongly disagree) to 3 (strongly agree), and with 0 representing “neither agree not disagree.”

**Table 3:** Measures used to assess judgments about belief, domain, and epistemic commitments in Experiment 2

Belief‐threat items	
1. *Threat*	“Saying ‘it's a mystery/it's unknown’ when asked why and how some things happen, is threatening to scientific/religious belief.”
2. *Importance*	“Saying ‘it's a mystery/it's unknown’ when asked why and how some things happen, makes scientific/religious belief seem less important.”
3. *Value*	“Saying ‘it's a mystery/it's unknown’ when asked why and how some things happen, makes scientific/religious belief seem less valuable.”
4. *Questioning*	“Saying ‘it's a mystery/it's unknown’ when asked why and how some things happen, makes one question scientific/religious belief.”
Domain‐threat items	
5. *Threat*	“Saying ‘it's a mystery/it's unknown’ when asked why and how some things happen, is threatening to science/religion.”
6. *Importance*	“Saying ‘it's a mystery/it's unknown’ when asked why and how some things happen, makes science/religion seem less important.”
7. *Value*	“Saying ‘it's a mystery/it's unknown’ when asked why and how some things happen, makes science/religion seem less valuable.”
8. *Value*	“Saying ‘it's a mystery/it's unknown’ when asked why and how some things happen, makes one question science/religion.”
Expert‐epistemic items	
9. Truth	“A/B values truth above all.”
10. Knowledge	“A/B is a knowledgeable person.”
11. Curious	“A/B is a curious person and always wants to know more.”

These three phases were subsequently repeated for the second domain. Data from a given domain were excluded if the participant did not pass the attention check (“for this item, please select the number between 3 and 1”) corresponding to that domain.

At the end of the experiment, participants completed a brief demographics survey where they were asked about gender, age, income, education, religiosity, and religious affiliation.

## 8 Results

The main goals of our analyses were to ask whether *unknown* versus *mystery* reflect different epistemic commitments, and whether they are differentially threatening across domains. Below, we present analyses that reflect each of our two main predictions. As in Experiment 1, we first included qualifying beliefs from each domain in our analyses (scientific beliefs: *N* = 894, religious beliefs: *N* = 713) and then repeated the analysis with participants who had qualifying beliefs from both domains (*N* = 588).

### 8.1 Epistemic commitments

**Fig. 3:**
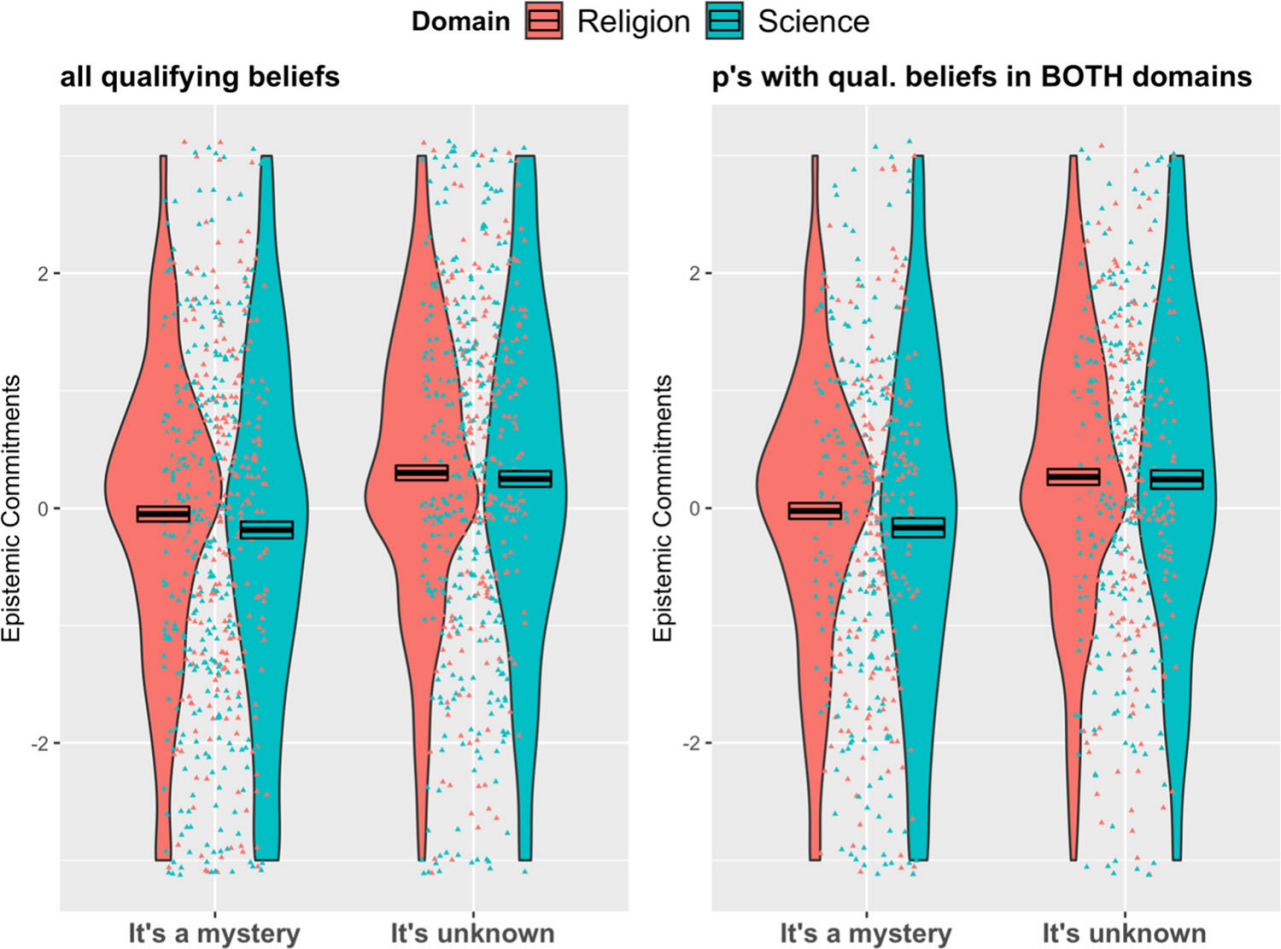
Experiment 2–The effect of Ignorance Type on the attribution of epistemic commitments by Domain, both when including all qualifying beliefs (left panel) and only participants with qualifying beliefs in both domains (right panel). Note: “0” represents “neither agree nor disagree.” Negative numbers indicate *lower attribution* of commitments (curiosity, knowledgeability, and valuation of truth) and positive numbers indicate *higher attribution* of commitments. Points represent jittered data points. Boxes represent average scores––black line in the middle––and +/– 1 SEM.

Our first prediction was that an expert's ignorance in the form of *unknown* (vs. *mystery*) would be seen as more reflective of epistemic commitments. To test this, we first created a composite Epistemic Commitments score based on the average ratings for “curious,” “knowledgeable,” and “valuing truth” (items 9–11 in Table 3). Based on pilot testing, we expected these items to form a single reliable construct, and reliability was indeed high (*α* = 0.87). We conducted a mixed‐effects linear regression model (using the *lme* function) on this composite score with Domain (Religion, Science), Ignorance Type (Unknown, Mystery), and their interaction as predictors, defining a random intercept accounting for participant‐level variability in ratings across Domains. This model revealed a main effect of Ignorance Type (*B* = 0.36, *SE* = 0.10, *t* = 3.66, *p* < .001, *CI* [0.16, 0.54]): when the expert indicated that the answer is unknown, participants attributed higher levels of curiosity, knowledgeability, and valuation of truth than when the expert indicated that the answer is a mystery. Neither the main effect of Domain nor the interaction between Domain and Ignorance Type was significant (*B* = –0.14, *SE* = 0.07, *t* = –1.89, *p* = .06 and *B* = 0.10, *SE* = 0.10, *t* = 0.96, *p* = .34, respectively; see Fig. 3). The pattern remained the same when we restricted analysis to participants with qualifying beliefs in both domains.

### 8.2 Threats to belief and domain

Our second prediction was that *mystery* (vs. *unknown*) would be perceived as more threatening to scientific beliefs and science. We also expected that this difference between *mystery* and *unknown* would be attenuated or reversed for religious beliefs and religion. To test these predictions, we first created a “threat” composite score based on ratings of value, importance, threat, and questioning for both beliefs and the domain as a whole (items 1–8 in Table 3). Based on pilot testing, we expected these items to form a single reliable construct, and reliability was indeed very high (*α* = 0.95).

**Fig. 4:**
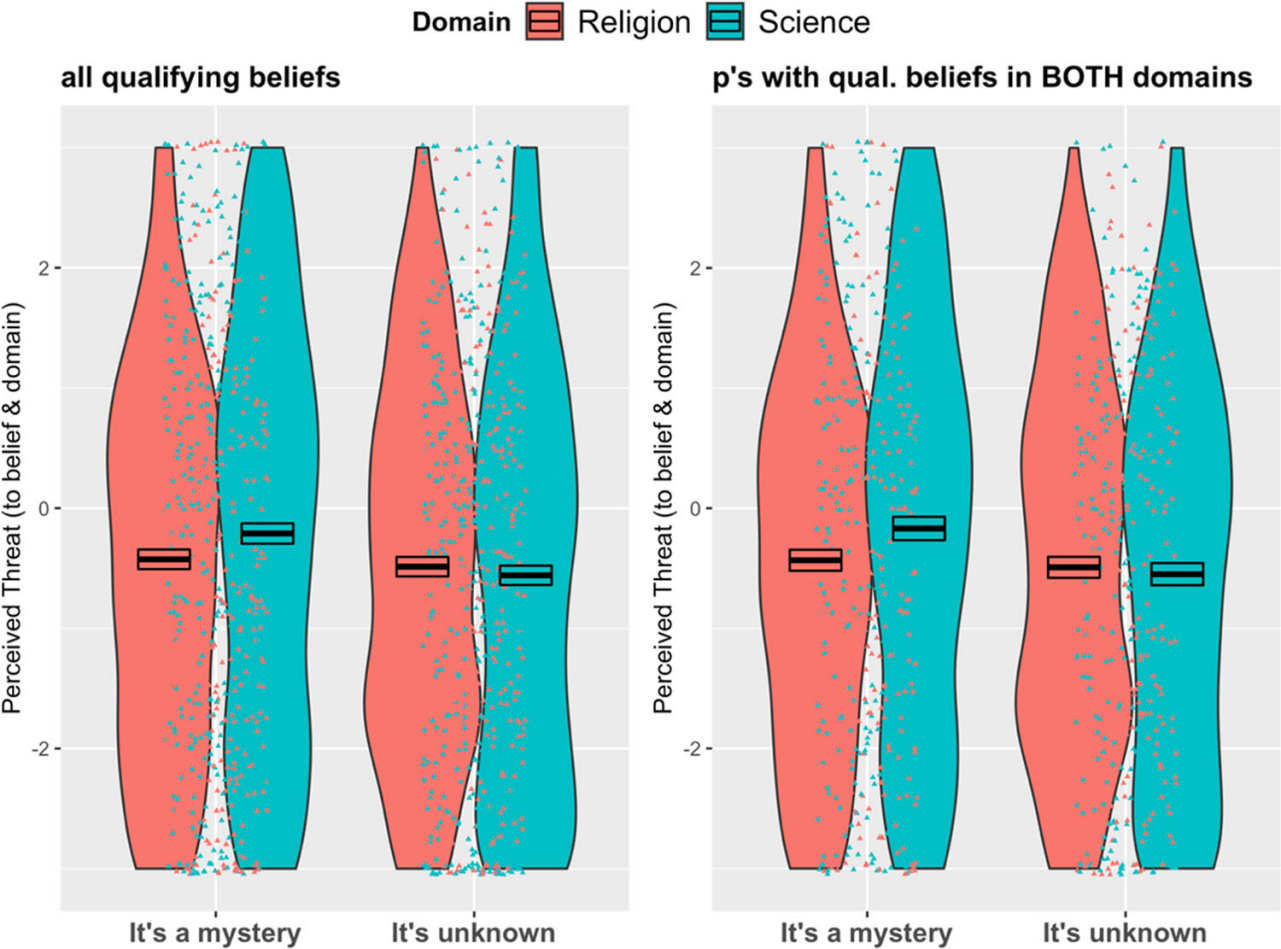
Experiment 2–Interaction effect between Domain and Ignorance Type on Threat composite score, both when including all qualifying beliefs (left panel) and only participants with qualifying beliefs in both domains (right panel). Note: “0” represents “neither agree nor disagree.” Negative numbers indicate less threat and positive numbers indicate more threat. Points represent jittered data points. Boxes represent average scores––black line in the middle––and +/– 1 SEM.

We then ran a mixed‐effects linear regression model on the Threat composite score with Ignorance Type, Domain, and their interaction as predictors. The model included a random intercept to account for within‐participant variability across Domains. We first ran this model on all qualifying beliefs and then only on data from participants with qualifying beliefs from both domains. Both models showed a significant main effect of Domain (*B * = 0.23, *SE * = 0.10, *t * = 2.10, *p* = .02, *CI *[0.03, 0.42], and *B * = 0.26, *SE * = 0.11, *t * = 2.47, *p* = .01, *CI *[0.05, 0.47], respectively) with greater threat from ignorance to science than to religion. The main effect of Ignorance Type was not significant in either model (*B * = –0.05, *SE * = 0.12, *t * = –0.43, *p* = .66, and *B * = –0.06, *SE * = 0.13, *t * = –0.46, *p* = .65, respectively) but the predicted interaction between Ignorance Type and Domain was (*B* = –0.30, *SE* = 0.14, *t* = –2.10, *p* = .036, *CI* [–0.57, –0.02] and *B* = –0.32, *SE* = 0.15, *t* = –2.12, *p* = .034, *CI* [–0.62, –0.02], respectively; see Fig. 4). We followed up this interaction by analyzing effects of Ignorance Type within each domain. For science, we found the predicted main effect of Ignorance Type such that mystery posed more threat as compared to unknown (*B* = –0.35––Mystery as reference, *SE* = 0.12, *t* = 2.90, *p* = .003, *CI* [–0.58, –0.12], and *B* = –0.38, *SE* = 0.13, *t* = –2.83, *p* = .005, respectively, for participants with qualifying scientific beliefs and those with qualifying scientific *and* religious beliefs). For religion, we found no significant effect of Ignorance Type (*B* = –0.06, *SE* = 0.11, *t* = –0.53, *p* = .60, and *B* = –0.06, *SE* = 0.12, *t* = –0.48, *p* = .63, respectively, for participants with qualifying religious beliefs and those with qualifying religious *and* scientific beliefs).

## 9 Discussion

Experiment 2 found that across the domains of both science and religion, an expert's ignorance was taken to indicate subtly different epistemic commitments depending on whether that ignorance was expressed as *unknown* or *mystery*. Specifically, when an expert said “It's unknown,” participants inferred greater curiosity, knowledgeability, and valuation of truth than when the expert said “It's a mystery.” This is consistent with the findings from Experiment 1 in that *unknown* (vs. *mystery*) was more strongly associated with science, and science was more strongly associated with epistemic goals and values (namely evidence and inquiry). However, it goes beyond Experiment 1 in demonstrating an experimental effect of ignorance type across domains, and in linking ignorance type to epistemic commitments more directly.

Experiment 2 also found differences in the role of ignorance across domains. Overall, participants tended to judge that a statement of ignorance was not threatening to the corresponding belief nor to its domain. However, they were least likely to dismiss ignorance as a threat when that ignorance came in the form of mystery in the domain of science. In the domain of science, “it's a mystery” was judged more threatening than “it's unknown.” In the domain of religion, no such difference was observed. These findings are consistent with the idea that because science has epistemic aims, forms of ignorance that are less aligned with epistemic aims are more threatening––they suggest a form of incongruity or failure. In Experiment 3, we further test effects of different types of ignorance across domains.

## 10 Experiment 3

Experiment 2 found that affirmations of ignorance were judged more threatening to science and scientific belief than to religion and religious belief, especially when ignorance took the form of *mystery* versus *unknown*. In Experiment 3, we tested an implication of this result: that ignorance about “how and why” some scientific proposition is the case should reduce confidence in the truth of that scientific proposition, with weaker (or absent) effects of ignorance on confidence in religious propositions. So, for example, learning that it is unknown (or a mystery) how and why the moon causes the tides should decrease confidence in the claim that the moon causes the tides, whereas learning that it is unknown (or a mystery) how and why God answers prayers should decrease confidence that God answers prayers to a smaller extent (or not at all). This result would extend the effect of ignorance on perceived threat to a belief or domain found in Experiment 2 to the much more personal currency of confidence in one's own beliefs. Following Experiment 2, we also predicted that for science, ignorance expressed as *mystery* (vs. *unknown*) would reduce confidence more strongly.

The procedures, predictions, and analyses for Experiment 3 were preregistered and are available in OSF at (https://osf.io/fh7ex/). A copy of the survey and data are available at (https://osf.io/jgykh/).

## 11 Method

### 11.1 Participants

Participants were 1004 adults recruited on Prolific (540 self‐identified as a woman, 440 as a man, 22 as nonbinary, and 2 as “other”; *M*_Age_ *=* 37 years, *SD*_Age_ = 14 years). Of these, 42% identified as Christian and 25% as Atheist, with the remaining 33% including “other” (13%), “Spiritual” (10%), and other religious affiliations––Buddhist, Jewish, Hindu, Muslim—as well as combinations of two or more affiliations (10%). Participation in all studies was restricted to Prolific workers in the United States who had not participated in any related studies, and who had an approval rating of at least 95% based on at least 100 prior tasks. An additional 251 participants were excluded from analyses because they did not meet criteria for belief generation in either domain (i.e., religious or scientific beliefs; *N* = 92), as detailed below, and/or because they did not pass attention checks in blocks where they did meet criteria for belief generation.

### 11.2 Procedure

Participants completed all procedures online using Qualtrics Survey Software (see OSF page for the survey). Each participant first consented to participate and pledged to pay attention and answer questions carefully. The introduction to the task was the same as that of Experiment 1, encouraging participants to think of a belief that they hold, but for which they do not know “the how and why.” Also as in Experiment 1, participants then completed a brief training on how to report the “content” of beliefs, and if they could generate a belief that met our requirements, they were asked to type its content into a text box. If they indicated that they could not think of such a belief in a given domain, they moved on to the same question for the other domain (order counterbalanced).

Once participants produced the content of a belief that met the requirements in either domain, the content of the belief was reproduced on a new page and they were asked “how confident are you that [content of belief]?”. Participants answered this “Confidence‐pre” measure on a scale of 1–7, with 7 representing “completely confident” and 1 representing “not so confident.” On the next page, they were asked to report the likelihood that an expert knows the “how and why” pertaining to the belief (Expectation of Knowledge: “even though you do not know how and why [belief], how likely do you think it is that experts do?”). This question was answered on a 1–7 scale, with 1 representing “not likely at all” and 7 representing “very likely.” We included this question to rule out a plausible interpretation of our predicted pattern of results: that participants expect the answers to scientific questions to be known, and the answers to religious questions to be unknown, such that a profession of ignorance is more challenging to the former only because it violates expectations. Measuring Expectation of Knowledge allowed us to account for such expectations in our statistical analyses.

After participants rated their level of confidence and Expectation of Knowledge with respect to their own belief, they moved on to a phase like that of Experiment 2. They were first introduced to an expert (“imagine someone who is an expert when it comes to scientific [religious] questions and whom you trust. This expert also believes that [content of participant generated belief]”). They were then asked to imagine that when asked how and why [content of belief] happens, the expert responds with either “it's a mystery” or “it's unknown” (between‐subjects). Participants were then encouraged to take a moment to think about the implications of the expert's response and to answer the following questions based on this response. Immediately after, they were asked to rate their level of confidence in the belief again (Confidence‐post).

Before moving on to the next block, participants also responded to an attention check (“for this item, please select the number on the scale that is greater than two but less than four”). If participants failed the attention check from a given block corresponding to belief from one of the two domains, data from that domain were excluded from analysis.

At the end of the experiment, participants completed a brief demographics survey where they were asked about gender, age, income, education, religiosity, and religious affiliation.

## 12 Results

As in Experiment 1, we first verified that participants who reported scientific or religious beliefs that met our requirements in fact generated propositions from the corresponding domains. Two independent coders coded all beliefs as “domain appropriate” or not, and agreement was substantial for religious beliefs and moderate for scientific beliefs (religious beliefs: 95% agreement; *kappa* = 0.66, *SE* = 0.06, *p* < .001, *CI* [0.54, 0.77]; scientific beliefs: 98% agreement; *kappa =* 0.43, *SE* = 0.11, *p* = .007, *CI* [0.17, 0.69]^1.^), with disagreements resolved by a third coder. This resulted in excluding 59 beliefs as domain‐inappropriate for religion (e.g., “humans create babies”; vs. 559 as domain‐appropriate included in analyses of all qualifying beliefs), and 20 beliefs as domain‐inappropriate for science (e.g., “horoscopes are true on a daily basis”; vs. 867 as domain‐appropriate included in analyses of all qualifying beliefs). However, including these beliefs in the analyses that follow does not change the reported patterns. A complete list of all domain‐appropriate and domain‐inappropriate beliefs generated by participants is included on OSF (https://osf.io/cys7f/).

As in the two prior studies, we first conducted analyses on all qualifying beliefs and then restricted the same analyses to participants who generated qualifying beliefs in both domains (*N* = 433).

### 12.1 Change in confidence

**Fig. 5:**
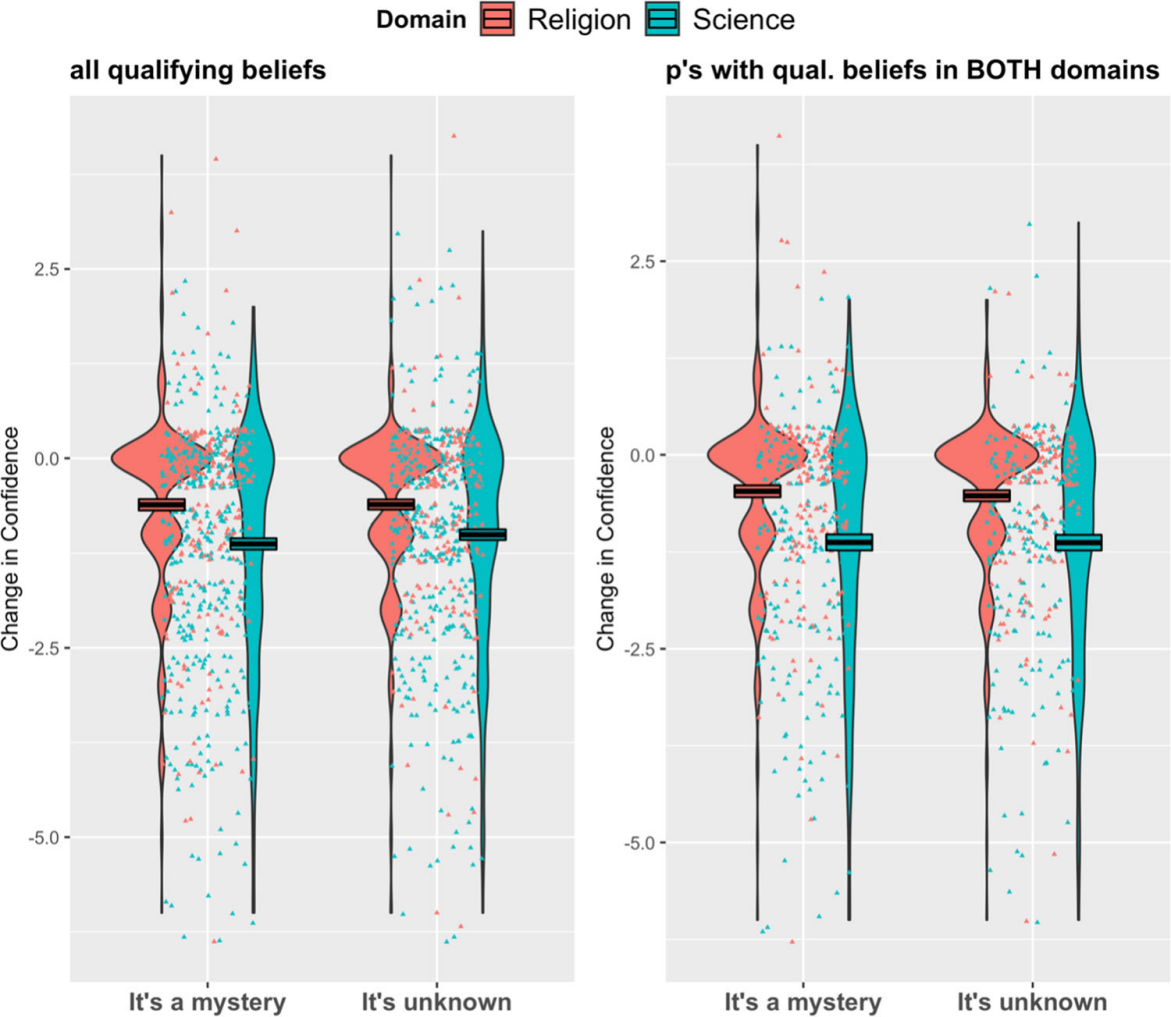
Experiment 3–Confidence Change scores as a function of Domain and Ignorance Type, both when including all qualifying beliefs (left panel) and only participants with qualifying beliefs in both domains (right panel). Note: Points represent jittered data points. Boxes represent average scores––black line in the middle––and +/– 1 SEM.

Our main prediction was that ignorance would result in a larger decrease in confidence for scientific beliefs than for religious beliefs, with a moderating effect of ignorance type (such that *mystery* was more threatening to science than *unknown*). To test this, we conducted a mixed‐effects linear regression model on Confidence Change (post – pre) with Domain, Ignorance Type, and their interaction term as predictors. We defined a by‐participant random intercept to account for within‐participant variability across the two domains. There was a main effect of Domain (*B* = –0.53, *SE* = 0.10, *t* = –5.51, *p* < .001, *CI* [–0.72, –0.34]), but there was no effect of Ignorance Type (*B* = 0.02, *SE* = 0.12, *t* = 0.22, *p* = .83), and no interaction between Domain and Ignorance Type (*B* = 0.10, *SE* = 0.14, *t* = 0.76, *p* = .45). The same pattern was found for participants who generated beliefs that met the requirement in both domains (Domain: *B* = –0.65, *SE* = 0.11, *t* = –5.98, *p* < .001; Ignorance Type: *B* = –0.06, *SE* = 0.13, *t* = –0.46, *p* = .65; Domain X Ignorance Type: *B* = 0.05, *SE* = 0.16, *t* = 0.35, *p* = .73). As shown in Fig. 5, confidence in scientific beliefs dropped more than confidence in religious beliefs, but this effect was not moderated by the type of ignorance professed by the expert.

To further probe the effect of Domain, we asked whether the difference in confidence change between scientific and religious beliefs could be an artifact of participants’ expectation about whether the answer to a given question is known (Expectation of Knowledge). We conducted a linear mixed‐effects model on Confidence Change with Domain as the predictor, while controlling for Expectation of Knowledge. For all qualifying beliefs and for participants with qualifying beliefs in both domains, Expectation of Knowledge significantly predicted Confidence Change (*B* = –0.07, *SE* = 0.02, *t* = –3.11, *p* = .002, *CI* [–0.12, –0.03] and *B* = –0.07, *SE* = 0.03, *t* = –2.45, *p* = .01, *CI* [–0.12, –0.01], respectively), but the main effect of Domain remained significant (*B* = –0.25, *SE* = 0.10, *t* = –2.52, *p* = .01, *CI* [–0.45, 0.06] and *B* = –0.42, *SE* = 0.12, *t* = –3.59, *p* < .001, *CI* [–0.64, –0.19], respectively). So, while answers to scientific questions were in fact judged more likely to be known than answers to religious questions, this difference does not fully account for the fact that confidence in scientific beliefs decreased more sharply in response to expert ignorance than did confidence in religious beliefs.

## 13 Discussion

Experiment 3 found a striking shift in participants’ own scientific beliefs in the face of ignorance. On average, participants reported that they would be less confident in their own reported scientific beliefs (e.g., “exercise keeps you healthy”) compared to their religious beliefs (e.g., “Christ will return to earth in the future”) if they heard a trusted expert affirm that the “how and why” of the belief is unknown or a mystery. Importantly, although participants judged scientific questions to be more “knowable” than religious questions, this did not explain the more sizeable decrease in confidence about scientific beliefs in the face of ignorance. Contrary to our predictions, an expert affirming “mystery” did not decrease confidence in scientific beliefs more than an expert declaring something “unknown.” Thus, Experiments 2 and 3 were consistent in finding weaker effects of ignorance in challenging religious belief than scientific belief, but unlike patterns in Experiment 2, Experiment 3 did not show a moderating role for ignorance type in this effect of domain.

## 14 General discussion

In both science and religion, (perceived) ignorance and (perceived) knowledge are arguably two sides of the same coin. Just as norms for knowledge might differ across domains, so too, might the role of ignorance: in science, ignorance about how and why something is the case often propels inquiry, but in religion, such ignorance may more readily be accepted as mystery. Across three experiments, we asked whether ignorance in the case of science is perceived differently from ignorance in the case of religion, and whether this difference reflects distinct profiles with respect to epistemic commitments and goals.

In Experiment 1, we found that scientific ignorance (i.e., not knowing how and why some scientific phenomenon is the case) is most often expressed as a personal unknown (“It's unknown to me”), whereas religious ignorance (i.e., not knowing how and why some religious phenomenon is the case) is more commonly expressed as a universal mystery (“It's a mystery”). Corroborating previous work on the epistemic qualities of science versus religion, Experiment 1 also documented stronger associations between scientific (vs. religious) questions and the perceived viability and value of inquiry (Liquin et al., 2020). We also predicted that even within each domain, expressions of ignorance in the form of “unknown” would be associated with a more epistemic profile than expressions of mystery. For instance, we expected that scientific “unknowns” would be perceived as more viable and valuable targets of inquiry than scientific “mysteries.” Despite the striking differences *across* domains in both expressions of ignorance and in the perceived value of inquiry, these predicted patterns of differentiation within domain were not observed.

In Experiment 2, we found that experts who reported that the answer to a scientific or religious question is unknown were perceived to be more knowledgeable, more curious, and more concerned with truth than were experts who reported that the answer to a scientific or religious question is a mystery. Thus, across domains, we did observe an association between expression of ignorance in the form of unknown (vs. mystery) and stronger expectations of adherence to epistemic values and epistemic achievements. Experiment 2 also found that ignorance is perceived to pose a greater challenge to science and scientific belief than to religion and religious belief. Similarly, Experiment 3 more strikingly found that participants’ confidence in their *own* scientific beliefs dropped more substantially compared to confidence in their religious beliefs after they were asked to imagine an expert who confessed ignorance. Thus, across both Experiments 2 and 3, we present evidence that ignorance poses a greater threat to scientific belief than to religious belief.

While the predicted patterns of variation across domains and forms of ignorance were consistent across studies, the interactions between domain and ignorance type were not. Notably, Experiment 2 found that “mysteries,” as compared to “unknowns,” were perceived as more threatening to science. However, this difference was not found in Experiment 3, which investigated a similar effect by measuring the decline in participants’ confidence in their own beliefs. We speculate that if this difference across experiments is reliable, it may have arisen from the different materials used. In Experiment 2, the set of beliefs that participants could select was restricted to five predesignated belief statements, all of which are prominent and common in public discourse (e.g., that the moon causes the tides; that CO_2_ emissions cause climate change; see Table 2). By contrast, in Experiment 3, participants generated their own beliefs, which tended to be more specialized and idiosyncratic (e.g., “oxygen makes cancer cells spread,” “not getting enough sleep can lead to obesity,” “muscles have a memory that is adaptive,” and “micro‐wounds end up healing the cells of the body”). It may be that learning that something is a mystery is especially threatening to scientific claims that are known to be elements of shared public discourse, versus those that are more personal.

With the exception of this inconsistency across Experiments 2 and 3, our findings are consistent across experiments, and also consistent with prior work in suggesting that within our largely Christian and U.S. adults sample, science and religion are indeed associated with different norms for knowledge and belief (e.g., Davoodi & Lombrozo, 2021), with correspondingly different attitudes toward inquiry (Gill & Lombrozo, 2019; Liquin et al., 2020). However, we go beyond this prior work in three important ways. First, we show that science and religion are associated with different forms of ignorance: personal unknowns versus universal mysteries (Experiment 1). Second, we show that these forms of ignorance are differentially associated with epistemic goals and norms: expressing ignorance in the form of “unknown” (vs. “mystery”) more strongly signals epistemic values and achievements (Experiment 2). Third, we show that universal ignorance (i.e., experts not knowing the answers) is perceived to be a greater threat to science and scientific belief than to religion and religious belief (Experiments 2 and 3).

A potential limitation of our work stems from the focus on “how and why” questions. Are the findings documented here likely to extend to other forms of ignorance and inquiry, such as ignorance concerning *whether* something is the case (e.g., Does the moon cause the tides? Does God answer prayers?). Gill and Lombrozo (2019) found that differences across science and religion in response to evidence‐seeking extended to the truth of claims themselves (e.g., whether the shroud of Turin is the burial cloth of Jesus), even when they did not concern the “how and why.” That said, we do expect boundary conditions on our effects. For instance, ignorance related to the performance of relevant duties is likely to be a call to action in either domain: How should a ritual be performed? How should a cell culture be maintained? Our expectation is that ignorance will prompt inquiry in either domain when it impedes the realization of the functions of belief in that domain, such that differences in the roles of ignorance stem from differences in the functional roles of the corresponding beliefs (see Davoodi & Lombrozo, 2021, for relevant discussion). Second, we expect that the threat of professed ignorance to belief (in Experiments 2 and 3) will only emerge for beliefs about which participants have some uncertainty. If a claim is firmly rejected (i.e., assigned zero confidence), ignorance will (trivially) have no effect. At the other extreme, if a claim is already accepted with extremely high confidence (e.g., that the dinosaurs became extinct, or that Jesus died for our sins), it seems unlikely that an expression of ignorance concerning “how and why” would have much, if any, effect, since high confidence was already achieved in the absence of such knowledge. In such cases, belief is likely to be based on sources of justification that do not depend on “how and why” (e.g., fossil evidence of dinosaur extinction and deference to religious authorities).

A second (and in our view, more substantial) limitation of this work is the exclusive focus on a largely Christian sample within the United States. It thus remains unclear whether the features associated with the domains of science and religion that we observe extend beyond this sample. In particular, it is plausible that adherents of other religious traditions differ strongly in their attitudes toward inquiry and mystery. As one example, world‐renowned Noble laureate in Physics and a devout Muslim, Abdus Salam, viewed his religious faith as an inspiration for his notable scientific findings. He believed that “the Holy Quran enjoins us to reflect on the varieties of Allah's created laws of nature,” and he saw his scientific career as doing just that (Lewis, 1980). Given this unique pattern of interplay between religious faith and scientific findings, one would expect religious “mysteries” to invite inquiry and a search for evidence. This would imply a similar pattern to our participants’ judgments of the implications of scientific unknowns for inquiry. Other religious traditions, such as Judaism, also promote inquiry concerning a variety of religious matters. In the words of Ismar Schorsch (2000), rabbi and professor of Jewish history, “the centrality of revelation never put a damper on the human right to question the divine.” Investigating differences across cultures (and across different religious communities within a given cultural context) is, therefore, an important direction for future research.

Even within Christianity itself, there is little doubt that interactions between cultural traditions, theological teachings, and personal factors will also lead to variability in attitudes toward inquiry about religious mysteries. For example, in contrast to the sentiment expressed by Monsignor Charles Pope (2013) to accept, respect, and stay away from scrutinizing religious mysteries, Christian priest John Van Sloten (2021) preaches about specific scientific findings and mechanisms (e.g., how the human knee or the human gut works) as a way to uncover the mysteries of creation and to express truths about God's mind. Specific cultural factors that might lead to this variability include the relationship between politics and religion. For example, in theocratic sociopolitical structures, religious mysteries may be more accepted and revered, whereas a democratic structure may encourage personal choice in seeking explanations to religious mysteries. Additionally, personal factors can impact one's attitude toward religious mysteries. For example, science curiosity (see Landrum, Hilgard, Akin, Li, & Kahan, 2016), or cognitive style (see Pennycook, Cheyne, Seli, Koehler, & Fugelsang, 2012; Shenhav, Rand, & Greene, 2012) may contribute to variability in inquisitiveness toward religious mysterious and explanation‐seeking behaviors. The interplay between relevant cultural and personal factors should be studied within and between particular religious traditions to better understand the extent to which our findings are generalizable, and more generally, whether and when a “scientific” attitude can be adopted towards religious content, or a “religious” attitude towards science.

A final limitation of our work worth highlighting relates to the limited range of epistemic attitudes we investigated. Specifically, while we show that objective, verifiable, and empirical ways of knowing are rated as less valuable and appropriate for religious beliefs compared to scientific beliefs, it is possible that within the domain of religion, some regard more subjective or personal experiences (such as miracles or mystical experience) as legitimate sources of evidence or justification for belief. In fact, in prior work, religious individuals have been documented to rely on religious sources or subjective experiences, such as what one feels in one's heart, as justifications for their religious beliefs (Metz et al., 2018; see also Davoodi et al., 2020). Thus, it is possible that with a broader range of measures, we would be able to identify different *forms* of evidence or inquiry associated with each domain. Relatedly, it might be important to ask whether there are further *kinds* of ignorance worth distinguishing. For example, might people approach scientific unknowns that are thought to be unknowable differently from those that are at present merely unknown? Are scientific matters that are thought to be beyond human comprehension (e.g., Chomsky, 2009) distinguished from those that we could comprehend but never know, such as the number of grains of sand in the world (e.g., Kominsky et al., 2016)?

These are important questions to raise within the broader project of characterizing the varieties of ignorance that shape cognition.

Our findings also raise new questions for science communication and scientific education. As noted in the introduction, recognizing ignorance and uncertainty is an inevitable and invaluable part of the scientific process (Firestein, 2012). The fact that scientific ignorance is sometimes regarded as threatening to science or scientific belief is, therefore, a potential concern for public understanding and acceptance of science. Prior work––for instance, in the context of risk communication and climate change––has investigated public responses to scientific uncertainty (Gustafson & Rice, 2020), including different ways in which uncertainty can be conveyed. It is striking that a subtle change in the linguistic expression of ignorance—from “It's unknown” to “It's a mystery”––has reliable (if small) effects on belief. Further research on expressions of ignorance and uncertainty is thus likely to play an important role in scientists’ and policy‐makers’ ability to craft effective messages about scientific content.

Reducing our ignorance about ignorance may seem like a roundabout way to get at the nature of epistemic commitments and intuitive theories of knowledge, but it is a powerful one: beliefs about what we can and should know shape decisions at all scales, from the questions we ask of others and ourselves to the funding policies we are likely to support. Focusing on the domains of science and religion helps reveal the systematic links between ignorance, inquiry, and belief, and the viability and value of investigating the psychology of what we do not know.

### Open Research Badges

This article has earned Open Data and Open Materials badges. Data and materials are available at https://osf.io/c9qf7/.

## Note

1. Although reliability was very high, Kappas are low due to a problem named “the paradoxes of kappa” (Yarnold, 2016).
